# Aerobic exercise and cognitive function in chronic severe traumatic brain injury survivors: a within-subject A-B-A intervention study

**DOI:** 10.1186/s13102-024-00993-4

**Published:** 2024-09-27

**Authors:** Lidia Pérez López, Margalida Coll-Andreu, Meritxell Torras-Garcia, Manel Font-Farré, Guillermo R. Oviedo, Lluis Capdevila, Myriam Guerra-Balic, Isabel Portell-Cortés, David Costa-Miserachs, Timothy P. Morris

**Affiliations:** 1 Department of Psychobiology and Methodology of Health Sciences, Institute of Neurosciences, Universitat Autònoma de Barcelona. Bellaterra (Cerdanyola del Vallès), Barcelona, Spain; 2 Department of Physical Activity and Sport Sciences, Faculty of Psychology, Education and Sport Sciences (FPCEE), University Ramon Llull, Císter 34, Barcelona, 08022 Spain; 3 Sport Research Institute, Autonomous University of Barcelona, Bellaterra (Cerdanyola del Vallès), Barcelona, Spain; 4 Department of Physical Therapy, Movement and Rehabilitation Sciences, Northeastern University, 360 Huntington Avenue, Boston, MA 02115 USA; 5 Center for Cognitive and Brain Health, Northeastern University, 360 Huntington Avenue, Boston, MA 02115 USA; 6 Department of Kinesiology, Mississippi State University, Mississippi State, USA

**Keywords:** Traumatic brain injury, Cognitive function, Physical exercise, Physical activity

## Abstract

**Background:**

Following acute and sub-acute rehabilitation from severe traumatic brain injury (TBI), minimal to no efficacious interventions to treat ongoing cognitive deficits are available. Aerobic exercise is a non-invasive behavioral intervention with promise to treat cognitive deficits in TBI populations.

**Methods:**

Six individuals, aged 24–62 years, with chronic (> 8 months since injury) severe (Glasgow Coma Scale of 3–8) TBI were recruited from two outpatient rehabilitation centers. In an A-B-A study design, 20-weeks of supervised aerobic exercise interventions were delivered three times per week (phase B) in addition to participants typical rehabilitation schedules (phases A). The effect of phase B was tested on a trail making test part B (primary outcome measure of executive function) as well as objective daily physical activity (PA), using both group level (linear mixed effect models) and single subject statistics.

**Results:**

Five of six participants increased trail-making test part B by more than 10% pre-to-post phase B, with three of six making a clinically meaningful improvement (+ 1SD in normative scores). A significant main effect of time was seen with significant improvement in trail-making test part B pre-to-post exercise (phase B). No significant effects in other planned comparisons were found. Statistically significant increases in daily moderate-to-vigorous PA were also seen during phase B compared to phase A with three of six individuals making a significant behaviour change.

**Conclusions:**

The addition of supervised aerobic exercise to typical rehabilitation strategies in chronic survivors of severe TBI can improve executive set shifting abilities and increase voluntary daily PA levels.

**Trial registration:**

Retrospective trial registration on July 11 2024 with trial number: ISRCTN17487462.

**Supplementary Information:**

The online version contains supplementary material available at 10.1186/s13102-024-00993-4.

## Background

Sustained deficits in executive functions, attention, memory, and processing speed are common following severe traumatic brain injury (TBI) and are a primary driver of poor quality of life and persistent functional disability in TBI survivors [1]. Around 65% of people who suffer moderate or severe TBI develop chronic cognitive deficits [2]. Yet, following acute and sub-acute rehabilitation, individuals re-integrating into community settings are faced with limited resources and no accessible or efficacious interventions to treat debilitating cognitive deficits. Long-term behavioural strategies such as lifestyle interventions could significantly benefit individuals with lasting TBI-related deficits [3], but limited evidence of their effect exists [4].

In non-injured adults, aerobic physical exercise has been shown to affect broad domains of cognitive functions, with the largest effect sizes being seen on executive functions [5–7]. Physical activities are often used in within sub-acute rehabilitation settings, but limited high quality evidence exists of how regimented physical exercise regimes can affect cognition in the chronic stages of recovery from moderate-to-severe TBI [4]. Prior human studies of physical exercise on cognitive function in TBI have included both heterogenous populations and recovery stages (mix of acute and chronic), short-term interventions (four to 12 weeks), retrospective study designs and single-arm studies [8–12]. Nevertheless, in several of these studies, improvements in cognitive function were reported, including improvements in executive functions [9]. Additionally, alternative treatments are currently being tested and significant improvements in executive functions have also been reported following invasive therapeutic interventions such as thalamic deep-brain stimulation in six individuals with chronic moderate-to-severe TBI [13]. While such invasive intervention shows promise for chronic severe TBI survivors, if similar gains can be achieved with non-invasive interventions, then more individuals living with chronic cognitive deficits from injury would benefit from accessible, economic and safe interventions.

An additional consideration with behavioural lifestyle interventions is the ability of individuals with executive deficits to voluntarily engage in the healthy behaviour outside of the prescribed intervention sessions. Executive functions, cortical brain regions underpinning executive functions and psychosocial constructs such as exercise self-efficacy are related to voluntary engagement in physical activity and exercise in non-injured adults [14–16]. Exercise interventions, even in sub-acute moderate-to-severe TBI have been shown to be feasible [10, 17], but survivors of TBI report significant barriers to participation in physical exercise, such as a lack of motivation, a lack of physician guidance, cost of programs and a lack of knowledge about how to exercise [18, 19]. Importantly though, if given certain resources (e.g., free access to local gymnasiums), community-dwelling individuals with moderate-to-severe TBI will adhere to physical exercise [20]. However, whether enrolment in a structured physical exercise intervention alone promotes adoption of physical activity outside of the intervention sessions in chronic severe TBI survivors is unknown. If individuals make a behaviour change because of participation of the structured intervention, then the long-term benefits of this intervention are increased.

Compared to currently non-effective pharmacological agents or promising invasive interventions such as deep brain stimulation, aerobic physical exercise represents an accessible, non-invasive and long-term behavioural intervention that can potentially treat executive function deficits in individuals with chronic severe TBI. Therefore, this study had two aims; (1) to test the effect of a 20-week aerobic physical exercise intervention on executive function in individuals with chronic severe TBI and (2) test the effect of the intervention on voluntary engagement in daily physical activity levels outside of the intervention sessions. We tested these aims using a within-subjects A-B-A study design with phase B consisting of 20 weeks of aerobic physical exercise sessions delivered three-times per week. We hypothesized that executive function performance would increase with the inclusion of 20-weeks of aerobic exercise (pre-to-post phase B) and that no changes would be evident between the other phases.

## Methods

Preparation of this manuscript was performed under CONSORT checklist guidelines for reporting results of clinical trials.

### Participants

Participants were recruited from two ambulatory neurorehabilitation centres between July 1 2019 and January 31 2020. All patients from both centres (as such, the resultant sample size is considered a pragmatic sample size and no formal calculations were performed) who met the following inclusion criteria were invited to participate in the study: (1) a severe TBI, defined as a score between 3 and 8 in Glasgow comma scale at time of injury; (2) a minimum of 8 months since injury, to capture community-dwelling individuals living at home; (3) aged between 18 and 65 years; (4) lack of medical contraindications to engage in physical exercise of moderate or vigorous intensity; (5) preserved communication abilities to perform neuropsychological assessments, and to give informed consent. Eight patients fulfilled these criteria two of whom declined to enrol in the study. The final sample consisted of 6 individuals (5 male, 1 female). This project was approved by the ethics committee for animal and human experimentation of the Autonomous University of Barcelona (CEEAH 4658). All the participants received verbal and written information about the study and signed informed consent. All measures were taken to preserve the confidentiality of the individuals’ identity.

### Study design

**Fig. 1:**
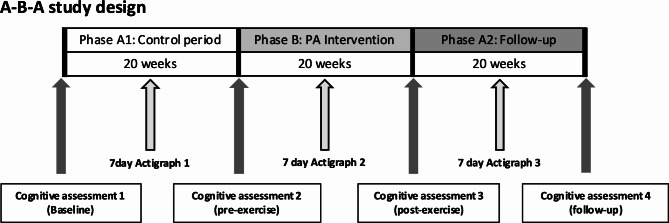
Study design followed an A-B-A design, whereby phase B consisted of 20-weeks of supervised aerobic exercise sessions delivered three-times per week. Wrist-worn physical activity measurements were collected for seven consecutive days during each study phase. Cognitive function assessments were completed four times before and after each phase

This was a single-arm within-subject study that followed an A-B-A design (Fig. 1). Three phases (20 weeks each) were designed as follows; Phase A1: Ongoing individually tailored outpatient neurorehabilitation (see Table 1 for a complete description); Phase B: tailored outpatient neurorehabilitation plus scheduled aerobic physical exercise (three sessions per week, 30 min/session); Phase A2: Tailored outpatient rehabilitation without the physical exercise intervention. The closure of the rehabilitation centres in response to the first wave of the covid-19 pandemic reduced Phase B to 17 weeks in five participants and online rehabilitation was provided to the participants in phase A2. Cognitive function was assessed at the beginning and end of each study phase. Daily physical activity levels were measured for seven consecutive days in each phase of the study.

### Aerobic exercise intervention (phase B)

The aerobic exercise intervention was delivered in-person within each rehabilitation centre using either a cycling rehabilitation trainer (MOTOmed Viva 2 leg trainer; RECK-technik GmbH & Co.KG; Betzenweiler, Germany) for two participants with motor impairment, a stationary cycle ergometer (Decathlon; Domyos Essential) or combined the use of a treadmill (Domfit F1. BH Fitness; Álava, Spain) and a MOTOmed. Each session (~ 30 min) consisted of a 5-minute warm up, 20 min of continuous exercise and 5-minute cool down. Resting HR and blood pressure were recorded before and after each session with the use of a hand-held sphygmomanometer (Omron electronics; Barcelona; Spain). The intensity of exercise was progressively increased with a target heart rate intensity zone of 60–80% heart rate reserve (HRR). This target heart rate corresponded to a moderate-to-vigorous intensity and was chosen based on World Health Organization Guidelines on physical activity [21] and prior exercise studies in TBI populations [4]. The corresponding heart rate in beats per minute was calculated using the Karvonen equation ([220-age]-resting heart rate x intended % HRR + resting heart rate) set from the 3rd week onwards. Intensity zones were re-calculated every four weeks to account for any changes in resting heart rate. Heart rate was continuously recorded during the exercise sessions by means of a wrist pulsometer (Polar M430; Polar Electro; Kempele, Finland). Participants were asked to rate their perceived exertion and comfort/distress every five minutes using Borg’s scale (6-20) of perceived exertion and a visual analogue scale, respectively.

### Primary outcomes: executive function

We defined our primary outcome measure in this analysis as trail making test part B (TMT-B), to be able to make direct comparisons with prior exercise studies [9] and alternative treatments for executive function deficits in moderate-to-severe traumatic brain injury [13]. Trail making B is an executive control measure testing working memory and set shifting abilities [22–24]. Using pencil and paper, participants were instructed to draw a connecting line between consecutive ordered circles containing alpha-numeric symbols, as fast as possible while maintaining accuracy. If an error occurred (connecting the circle containing “2” to circle “F” for example, instead of circle “B”), participants were instructed to return to the last correct circle without stopping the clock. Given the severe deficits of the participants in this study, we did not limit their response time. Raw completion time scores are used in subsequent statistical analyses, and we also present percentage change and scaled normative scores to increase interpretability. Normalized scaled scores were calculated based on the Neuronorma project for the Spanish population [25] whereby the population mean is 10 and a 1 standard deviation change is equal to 3.

Several additional executive function assessments were collected as secondary executive function outcome measures, as executive functions are particularly vulnerable to injury and have been shown to be modulated by aerobic exercise in other populations. These included the Rey-Osterrieth complex figure test, including a copy phase (perceptual and motor capacity) and a memory phase (30 min later), measuring visuospatial memory [26] and The Card Sorting Test (CST), using the short-form of the Berg’s CST (Psychology Experimental Building Language -PEBL) (64 cards instead of the 128 used in the long form) [27], which assesses ability to change strategies based on the demands of the task as a measure of flexibility. All tests were administered by a trained neuropsychologist in-person.

### Secondary outcome: physical activity

Engagement in voluntary physical activity in daily life was recorded during three periods of seven consecutive days, in each of the study phases (see Fig. 1 for an illustration of the study design). Seven consecutive days were chosen to ensure that participants wore the accelerometer for at least ≥ 4 (week and weekend) days for ≥ 10 h/day of waking hours. The first recording took place on either week two or week three of the study period; the second recording was taken on either week fourteen or fifteen and the third, within one week of the end of the study period, shortly after the end of the covid-19 lockdown. To measure physical activity and sedentary behaviours, an Actigraph wT3X-BT accelerometer (Actigraph, LLC; Pensacola FL, USA) was worn on the waist during each day of the recording periods (the device was not worn at night nor during bathing or water activities and only one participant wore the device during the exercise sessions- these recordings were subsequently removed during data processing). Physical activity and sedentary behaviours were analysed with the following criteria: Raw accelerometer data were downloaded and successively converted into 10-s epochs. Six consecutive epochs were summed to obtain activity counts per minute (cpm). All data were downloaded and processed using the ActiLife software (Firmware 4.4.0, Actigraph, LLC; Pensacola FL, USA). Based on cpm, physical activities were classified into sedentary (less than 100 cpm); light (between 100 and 1951 cpm), moderate (between 1952 and 5724 cpm), vigorous (between 5725 and 9498 cpm) and very vigorous physical activity (9499 cpm and above). For our primary outcomes, percent times in light physical activity (100–1951 cpm), moderate to vigorous physical activity (MVPA; ≥ 1952 cpm) and sedentary time (< 100 cpm) were calculated based on total daily recording minutes.

### Statistical analysis

All statistical analyses were performed using Jamovi (The Jamovi project, 2023), a R-based software. Linear mixed effects regression models were used to test for changes in our primary outcome (TMT-B) over time (beginning of phase A1 [baseline], beginning of Phase B [pre-exercise], post-phase B [post-exercise], and post-phase A2 [follow-up]). Mixed effects models are the recommended statistical technique [28] for analyzing outcomes measured at repeated timepoints as they can properly account for correlation between repeated measures within subjects. Importantly, these models are suitable for small-N studies [29] and can be robust to violations of distributional assumptions [30]. Nevertheless, these assumptions were checked using Q-Q plots and residual vs. fitted plots which are shown in supplementary S5. Each outcome measure was included as a response variable, assessment time (four repeated measures) as a fixed effect and a participant specific random intercept. Age and years of education were included as covariates in the model. Three planned comparisons were performed to test the effect of both introducing the intervention during phase B and removing it for phase A2. These comparisons were (1) Pre-exercise phase B compared to baseline beginning of phase A1 (to minimize and test for practice effects), (2) post-exercise phase B compared to pre-exercise phase B (main effect of the intervention) and (3) post-phase A2 (follow-up) compared to post-exercise phase B (the effect of removing the intervention). These planned comparisons allow one to test the effect of the addition of the phase B intervention, whereby if the intervention has an effect, significant changes in outcomes would only be observed in the pre-to-post phase B comparison. Full model effect sizes are presented as marginal R squared. Planned comparisons of the mean change in the raw scores are presented with 95% confidence intervals and Bonferroni-corrected p-values, with significance set at p <  0.05. To minimize multiple comparison issues with a small sample size, for the secondary executive function outcomes (trail making test part A, Rey-Osterrieth complex figure test, Card sorting test) we only present effect sizes and 95% confidence intervals, which will aid in future hypothesis generation while minimizing the number of statistical inferences being made. To test for changes in voluntary physical activity and inactivity, data were analysed at the group level using similar linear mixed effects models as previously described with assessment times being reduced to the three (during phase A1, during phase B and during phase A2) assessment time points when Actigraph data were collected. Two planned comparisons to test for changes in PA as a function of phase B were conducted testing phase B to phase A1 and phase B to phase A2.

## Results

Table 1 provides descriptive and demographic details for each participant, including a description of their injury, major cognitive deficits, and individual tailored rehabilitation programs. The exercise intervention was well tolerated by all participants, and no adverse events were reported. A mean adherence rate to the intervention sessions during Phase B of 72.22 ± 18.70% was recorded (individual adherence data are presented in Table S1). Heart rate response to exercise was limited in the two individuals with motor impairments (P1 and P5) where mean time spent within the heart rate training zones during intervention sessions was minimal. Three participants spent > 50% of intervention sessions within the heart rate training zones and one participant spent ~ 25% of time within the heart rate training zones (Table S2).

**Table 1:** Participant characteristics and phase A description

Patient	Time since injury	Sex	Age	Cause of TBI	Injury severity	Main cognitive areas impaired at baseline	Other areas affected	Characteristics of individually-tailored outpatient neurorehabilitation
P1	16 years	Male	45	Work accident. Crushed by a falling wall	Severe. GCS: 4/15	Processing speed, selective and divided attention, perseverative errors and WM	Behavioral and motor deficits. Hemiparesis and dysarthria. A wheelchair user, but can stand up with the aid of crutches.	8 h/day, 5 days per week engaged multiple activities in an outpatient center (social activities, physiotherapy)
P2	8 months	Female	24	Motorbike accident. Head on collision with an oncoming van	Severe. GCS: 3/15 Comma (2 days). Required craniotomy to alleviate intracranial pressure. 3 months hospitalization	Phonological fluency, planning, categorization, processing speed, alternating attention, naming, immediate and delayed memory	Emotional deficits	3 sessions/week of neuropsychological rehabilitation at an outpatient center
P3	4 years	Male	43	Motorbike accident	Severe. GCS: 3/15. Comma state for 15 days post-TBI	Planning and WM	Low emotional inhibitory capacity. Presented with high level of social inclusion and participated in many community events	1 h of neuropsychological rehabilitation every two weeks. Volunteer at the rehabilitation center, helping to organize social activities
P4	22 years	Male	42	Motorbike accident	Severe. GCS: 4/15 Comma states for 15 days post-TBI	Phonological fluency, planning, categorization, processing speed, alternating attention, delayed memory and WM	Behavioral and motor sequelae. Hemiparesis. A wheelchair user, but can stand up with the aid of crutches. Presented with apathy and behavioral disinhibition	3 h/week of rehabilitation in an outpatient center (physiotherapy, social activities)
P5	15 months	Male	62	Work accident. A fall from four stories high	Severe. GCS: 4/15 1 month hospitalization. Comma state for 20 days post-TBI	Phonological fluency, planning, alternating attention, attention span, WM, immediate and delayed memory	Polytrauma. Behavioral and motor impairments.	1 h/week of neuropsychological rehabilitation in an outpatient center
P6	29 months	Male	56	Car accident. Truck collision	Severe. GCS: 5/15 3 months of hospitalization	Alternating attention, planning, categorization, immediate and delayed memory	Polytrauma. Behavioral and physical alterations. Exophthalmos, that was surgically remediated shortly after the beginning of the study	2 h of neuropsychological rehabilitation every 2 weeks at two different centers Discontinued tailored rehabilitation sessions during phase B

GCS: Glasgow Coma Scale, ≤ 8 = severe

### Cognitive function

**Fig. 2:**
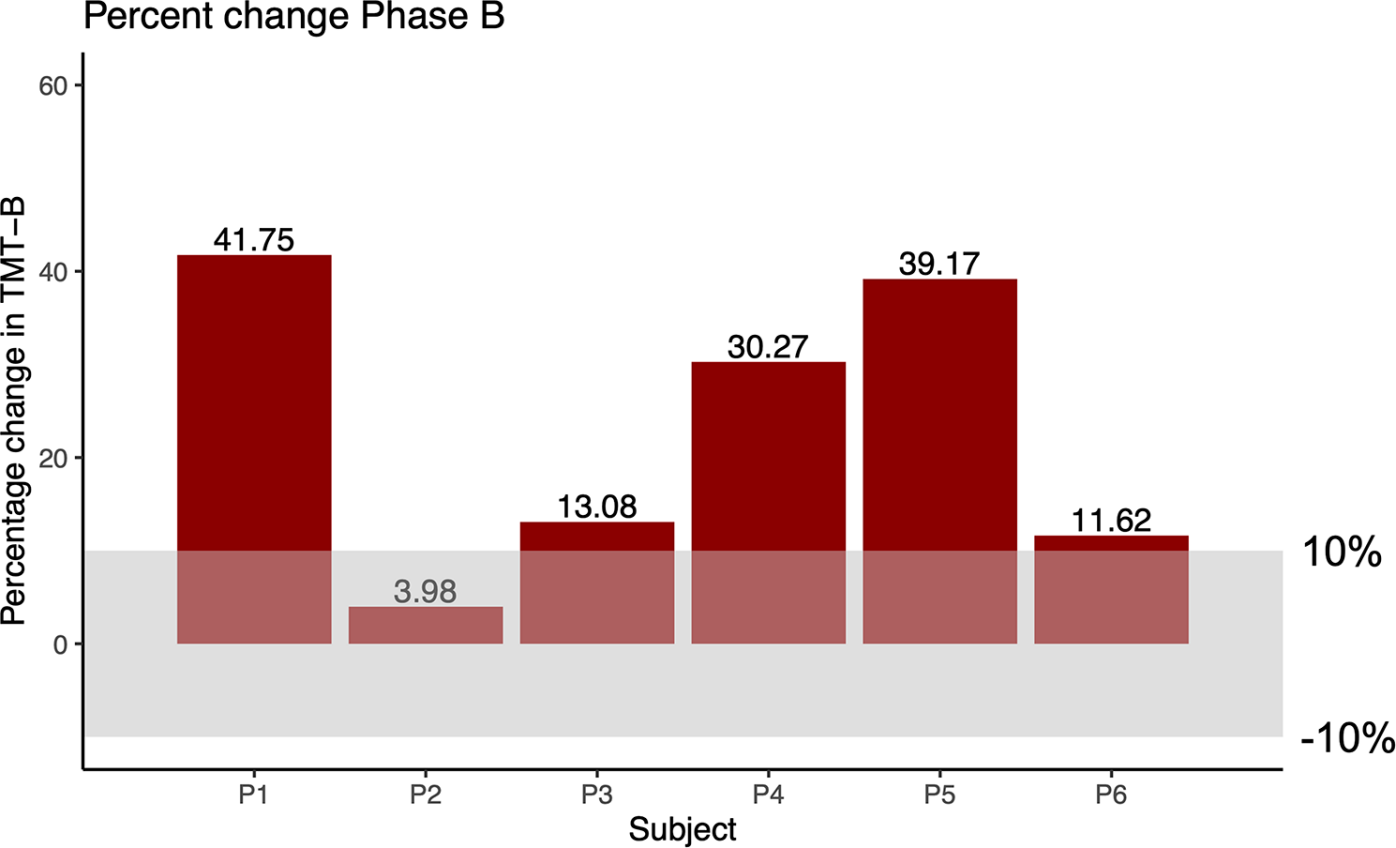
Individual percentage change in trail-making test B from pre-to-post exercise (phase B). All participants improved performance, with five of six increasing by more than 10%

Mean (± SD) raw scores, full model results and effect sizes for each cognitive test at each assessment time point are shown in Table 2. Five of the six participants improved TMT-B performance more than 10% pre-to-post exercise (Fig. 2) which equates to a 0.66 SD increase in one participant and a 1 SD increase in three participants (Table 3). At the group level, a significant main effect of time was found for TMT-B [F(3, 13.02) = 3.67; *P* = .041]. Planned comparisons showed significant improvements in TMT-B performance from pre-to-post phase B.

**Table 2:** Cognitive function outcomes

Test	Baseline Pre-phase A1 mean (range)	Phase A1 Pre-exercise mean (range)	Phase B Post-exercise mean (range)	Phase A2 Post-phase A2 mean (range)	Main effect of time	A1-Baseline Mean difference (95% CI)	B-A1 Mean difference (95% CI)	A2-B Mean difference (95% CI)
TMT-B (seconds)	302 (107, 500)	344 (130, 515)	244 (113, 311)	208 (130, 349)	F(3.13.02) = 3.67 *P* = .041 Marginal R^2^ = 0.082	42.17 (-21.8; 106.1)	-100.50* (-36.6; -164.4)	13.85 (-59.9; 87.6)
TMT-A (seconds)	95.7 (25, 235)	72.2 (28, 126)	77.8 (28, 129)	73.8 (21, 127)	F(3,15) = 0.51 Marginal R^2^ = 0.08	-0.17 (-0574; 0.232)	0.045 (-0.357; 0.449)	-0.12 (-0.526; 0.28)
ROCF-copy	35.2 (33, 36)	34.3 (34, 36)	34.3 (34, 36)	36 (36, 36)	F(3,15) = 5.56 Marginal R^2^ = 0.425	-0.83 (-1.77;0.104)	0 (-0.937; 0.937)	1.66 (0.729; 2.60)
ROCF-memory	19.1 (8.5, 35)	21.3 (10, 30)	19.2 (8.5, 28)	21.9 (9, 29)	F(3,15) = 0.92 Marginal R^2^ = 0.346	2.25 (-1.97; 6.47)	-2.166 (-6.38; 2.05)	2.75 (-1.47; 6.971)
CST Categories	3.83 (0, 8)	4 (1, 9)	3.50 (1, 9)	4.17 (1, 9)	F(3,15) = 0.49 Marginal R^2^ = 0.221	0.167 (-1.031; 1.36)	-0.500 (-1.697; 0.697)	0.667 (-0531; 1.86)
CST total errors	46.3 (25, 66)	46.7 (25, 63)	51.3 (18, 71)	39.8 (17, 58)	F(3,15) = 2.18 Marginal R^2^ = 0.299	0.333 (-8.53; 9.198)	4.667 (-4.20; 13.532)	-11.50 (2.637; 20.37)
CST Perseverative errors	18.2 (0, 34)	26.3 (0, 44)	20.3 (0, 45)	19 (1, 44)	F(3,15) = 0.41 Marginal R^2^ = 0.148	8.167 (-7.77; 24.10)	-6.00 (-21.94; 9.94)	-1.33 (-17.27; 14.60)

TMT: trail making test. ROCF: Rey-Osterrieth complex figure test. CST: Card sorting task. Effect sizes (R2) are presented only for secondary outcomes. *: statistically significant after Bonferroni-correction for multiple comparisons

**Table 3:** Scaled scores for pre-to-post exercise change in trail-making test part B

Participant ID	Pre-exercise TMT-B scaled score	Post-exercise TMT-B scaled score
P1	2 (2.66 SD below the mean)	5 (increase by 1 SD)
P2	6 (1.66 SD below the mean)	6 (no change)
P3	9 (normal range)	10 (normal range)
P4	2 (2.66 SD below the mean)	5 (increase by 1 SD)
P5	2 (2.66 SD below the mean)	5 (increase by 1 SD)
P6	6 (1.66 SD below the mean)	7 (increase by 0.66 SD)

Scaled scores were calculated using the Neuronoma project for the Spanish population whereby the population mean is equal to 10 and a one standard deviation change is equal to three. Five of six participants were below the normal range at the beginning of the exercise intervention

### Physical activity and inactivity outside of the intervention sessions

A significant main effect of time was found for daily percent time spent in MVPA [F(_2,10_) = 2.43; *p* = .046, _marignal_R^2^ = 0.06). Planned comparisons showed that daily percent time spent in MVPA significantly increased in phase B compared to phase A1 (mean estimate increase: 1.958%, 95% CI: 0.624; 3.29–14.63 min, 95% CI 4.76; 24.50). No other significant main effects were seen for light physical activity or time spent sedentary (full model results are found in Table 4). Individual-level analyses of physical activity change are presented in Table S3.

**Table 4:** Daily physical activity

Actigraph measure	Phase A1 Pre-exercise	Phase B Post-exercise	Phase A2 Post-phase A2	Main effect of time	B-A1 Mean difference (95% CI)	A2-B Mean difference (95% CI)
% sedentary time	81 (6.56)	72.5 (11.32)	78.8 (6.66)	F(2,10) = 2.43 *P* = .13 Marginal R^2^ = 0.159	-8.45 (-16.26; -0.639)	6.30 (-1.51; 14.11)
% light physical activity	15.6 (6.14)	22.2 (12.75)	16.5 (4.66)	F(2,10) = 1.65 *P* = .24 Marginal R^2^ = 0.075	0.304 (-0.030; 0.639)	-0.202 (-0.536; 0.132)
% MVPA	3.45 (2.50)	5.44 (3.50	4.70 (3.55)	F(2,10) = 4.25 *P* = .046 Marginal R^2^ = 0.062	1.958* (0.624; 3.29)	-0.707 (-2.04; 0.627)

MVPA: moderate-to-vigorous physical activity. * indicates statistical significance after Bonferroni correction

## Discussion

In this study we found that the addition of a 20-week supervised aerobic physical exercise intervention significantly increased executive function in chronic survivors of severe TBI. In addition, we found that voluntary engagement in physical activity, specifically moderate-to-vigorous physical activity (MVPA), was increased during the intervention phase. Our results build on limited literature testing the effect of aerobic physical exercise on cognitive function in survivors of severe TBI, and provide evidence that aerobic exercise is a potentially efficacious treatment for chronic executive function deficits in this population.

Rich pre-clinical literature suggests that aerobic exercise is both neuroprotective and evokes mechanisms of neuroplasticity following TBI, resulting in significant improvements in cognitive function [31–38]. Nevertheless, promising pre-clinical models have not been completely or successfully translated into human clinical studies [4]. Two prior studies [8, 9] who used interventions of three and six months, respectively, did find improvements in set shifting performance and subjective cognitive complaints. Our results add to prior work using a more robust study design (compared to single arm studies) whereby compared to our no-intervention phase A, set shifting abilities were significantly improved when including 20-weeks of aerobic exercise delivered three-times per week to the participants’ rehabilitation. Importantly, five out of six participants increased performance by more than 10%, with four showing a population-based increase of around 1SD, which reflects an effect size that has been considered clinically meaningful in other population-normed studies [39]. Notwithstanding, multiple studies have shown no significant effects of aerobic exercise [10, 11, 40] on either objective cognitive tests (trail making test included) or cognitive scales such as the cognitive sub-scale of the functional independence measure. These prior studies implemented short-term interventions (4 to 12 weeks) and included heterogenous participants in terms of injury severity [10, 11, 40]. As such, it is possible that engagement in physical exercise for longer durations is necessary to result in significant improvements in cognitive function, which has also been shown to be the case in older adults with and without mild cognitive impairment [41]. Similarly, a recent meta-analysis of studies testing the effects of exercise on cognitive function in stroke patients, which are more common than in TBI populations, draws specific recommendations (at least 3 exercise sessions per week, with a duration between 30 and 60 min each), and reaching a total of 180 min per week after a time [42].

Currently, no pharmacological agent has been proven to treat cognitive deficits in TBI. As such, alternative treatments have been developed and tested with promising results. For example, Schiff and colleagues [13] tested the initial feasibility of thalamic deep brain stimulation to treat chronic cognitive deficits in individuals with moderate or severe TBI and reported significant improvements in set-shifting abilities (trail-making B). Another behavioural intervention, cognitive training, is ubiquitous in in-patient rehabilitation of TBI and has shown preliminary efficacy in improving performance on cognitive performance in brain injured populations [43, 44]. Our results suggest that the effects of exercise lie predominantly with executive set shifting abilities, consistent with prior studies in TBI [9]. However, in addition to the effects of cognitive function, prior work studying the effects of physical activity (of which physical exercise is a sub-component of) have shown large effects on quality of life [45] and overall morbidity and mortality [46, 47]. Therefore, this behavioural intervention can potentially have broad reaching health benefits.

Voluntary engagement in physical activity outside of an exercise intervention study is important for the long-term adoption of this healthy lifestyle behaviour. If individuals with TBI make a behaviour change during participation in prescribed physical exercise such that they engage in more voluntary physical activity and less sedentary behaviour, then the cumulative effect of such interventions would be increased. Barriers to participation in physical activity have been studied in TBI populations [18, 19], and specific TBI barriers exist. One barrier that is consistently reported is a lack of advice or information on where and how to exercise. In one prior study, providing free access to local gymnasiums, Devine and colleagues [20] showed that TBI survivors increased their voluntary physical activity. At the group level, our results suggest that when participants were engaged in supervised physical exercise, they too increased their voluntary physical activity habits, specifically the amount of time spent performing moderate-to-vigorous physical activity. It is possible therefore that in survivors of severe TBI, simply providing access and education on physical activity could be enough to increase voluntary participation in this lifestyle habit. Nevertheless, when looking at the individual physical activity data, only three of the six participants increased their physical activity. Consequently, individual behaviour change interventions are likely required to evoke behavioural change in every participant.

Our study has several limitations that should be considered when interpreting the results. The small sample size limits the generalizability of our findings, notwithstanding, the use of linear mixed effects models enhances confidence in the statistical analysis and our use of percentage change in phase B allows one to make comparisons with comparable invasive interventions. However, a properly powered randomized study with a comparator condition will provide concrete inferences on the effect of this intervention in this population. Phase A2 was confounded by the closure of the rehabilitation centres due to the lockdown imposed because of the Covid-19 pandemic which may have affected the data collected during phase A2, particularly the voluntary physical activity recordings. Notwithstanding, this confound does not limit our confidence when making inferences comparing phase B to phase A1. The use of a single primary cognitive outcome measure limits the ability to test for broad changes in cognitive function as a result of this intervention and future studies should be properly powered to detect changes across a variety of cognitive outcomes. We provide the effect sizes and mean changes for several other executive function tasks to increase future hypothesis generation but avoid making statistical inferences on these outcomes. Lastly, only three participants exercised within the heart rate training zones during the supervised intervention sessions which highlights the difficulty in thresholding exercise intensity in severe TBI patients, which is consistent with one prior study [17]. Notwithstanding, time spent in heart rate training zones was not reflective of the individual effect sizes on cognitive outcomes. Future exercise studies need to develop protocols to threshold the intensity of aerobic exercise in moderate and severe TBI populations. Studies should test if heart rate response to exercise is reduced in this population and the associated physiological mechanisms, or if the measurements used to threshold exercise are unreliable in this population.

## Conclusions

Supervised aerobic exercise is potentially effective at improving executive function deficits in chronic survivors of severe TBI. It is likely that sustained periods of exercise training are necessary to see beneficial effects. Simply engaging in supervised physical exercise may however be enough to encourage those with severe TBI to become more physically active in their daily life. More research is needed to discover the mechanisms of such a behaviour change.

## Electronic supplementary material

Below is the link to the electronic supplementary material.

Supplementary Material 1

Supplementary Material 2

## Data Availability

The dataset used in the analysis during the current study are available from the corresponding author on reasonable request.
