# Supplementary material for: Aerobic exercise and cognitive function in chronic severe traumatic brain injury survivors: a within-subject A-B-A intervention study
Source: BMC Sports Sci Med Rehabil. 2024 Sep 27;16:201. doi: 10.1186/s13102-024-00993-4 (PMC11438162; doi:10.1186/s13102-024-00993-4)
Supplement: Supplementary file 1 — Supplementary Material 1 [file 13102_2024_993_MOESM1_ESM.docx]

**Table S1**. Adherence to the exercise intervention.

| Participant | Number of sessions completed | Percent sessions (based on total number of sessions available until lockdown) |
| --- | --- | --- |
| 1 | 38 | 74.51 |
| 2 | 60 | 100 |
| 3 | 25 | 49.02 |
| 4 | 44 | 86.27 |
| 5 | 30 | 58.82 |
| 6 | 33 | 64.71 |

Mean adherence: 72.22% (18.70)

**Table S2.** Heart rate training zones and % change in raw TMT-B scores.

| ID | % time below target zone | % time within target zone | % change in raw TMTB score |
| --- | --- | --- | --- |
| 1 | 89 | 11 | -41.75 |
| 2 | 41 | 59 | -3.98 |
| 3 | 10 | 64 (26% above the target zone) | -13.08 |
| 4 | 76 | 24 | -30.27 |
| 5 | 100 | 0 | -39.17 |
| 6 | 17 | 63 | -11.62 |

Those who exercised less in the heart rate training zones mostly increased TMT-B performance pre-to-post exercise the most. It is possible that less intense exercise is more beneficial for cognitive function, however it is more likely that this pattern of improvement is due to the fact that those who improved the most during the intervention phase were those who had worse performance at the start of the intervention phase (see main table 3).

**Table S3.** Individual level physical activity monitoring data

| Participant | Variable | Tau-U (90% confidence interval) Phase B compared to Phase A1 | Tau-U (90% confidence interval) Phase A2 compared to Phase B |
| --- | --- | --- | --- |
| 1 | % sedentary  % Light physical activity  % MVPA | -1 (-1; 0.421); P=**.0045**  1 (0.42; 1); P=**.0045**  1 (0.42; 1) P=**.0045** | 1 (0.47; 1) P=**.0017**  -1 (-1; -0.47) P=**.0017**  -0.95 (-1; -043) P=**.0027** |
| 2 | % sedentary  % Light physical activity  % MVPA | -0.25 (-0.76; 0.26); NS  -0.21 (-0.72; 0.29); NS  0.07 (-0.436; 0.579); NS | 0.21 (-0.29; 0.72); NS  0.55 (0.026; 1); NS  0.30 (-0.219; 0.83); NS |
| 3 | % sedentary  % Light physical activity  % MVPA | -0.93 (-1; -0.42) P=**.0026**  0.93 (0.42; 1) P=**.0026**  0.53 (0.028; 1); NS | 0.78 (0.28; 1); P=**.011**  -0.82 (-1; -0.31); P=**.0078**  -0.27 (-0.77; 0.24); NS |
| 4 | % sedentary time  % Light physical activity  % MVPA | -0.014 (-0.69; 0.405); NS  0.28 (-0.26; 0.83); NS  0.19 (-0.36; 0.74); NS | -0.10 (-0.58; 0.38); NS  0.43 (-0.045; 0.92); NS  -0.02 (-0.57; 0.45); NS |
| 5 | % sedentary  % Light physical activity  % MVPA | 0.14 (-0.40; 0.69); NS  -0.14 (-0.69; 0.40); NS  0 (-0.55; 0.55); NS | -0.55 (-1; -0.026); NS  0.55 (0.026; 1); NS  0.31 (-0.22; 0.83) |
| 6 | % sedentary  % Light physical activity  % MVPA | -0.87 (-1; -0.26); P=**.017**  0.07 (-0.53; 0.66); NS  1 (0.39; 1); P=**.006** | 0.25 (-0.32; 0.84); NS  -0.2 (-0.78; 0.38); NS  0.08 (-0.49; 0.66); (NS) |

Single-subject analyses using Tau-U statistic found that three out of the six participants showed either a decrease of sedentary time, or an increase of physical activity during stage B, compared to baseline data, while no significant changes were detected in the other three participants. Tau-U analyses also indicated that, in two of these three individuals, the amount of sedentary time increased following cessation of the exercise intervention, and the amount of physical activity decreased again when comparing the follow-up data to the data recorded during the period of exercise intervention.

**Supplementary material S4** - Exercise intervention description

Exercise intervention sessions were completed on weekdays during normal business hours and the time of day was kept consistent for each participant across sessions. Sessions were individual with the same study team member (LPL) delivering each intervention session. Each participant used either a cycling rehabilitation trainer (MOTOmed Viva 2 leg trainer; RECK-technik GmbH & Co.KG; Betzenweiler, Germany) for two participants with motor impairment, a stationary cycle ergometer (Decathlon; Domyos Essential) or combined the use of a treadmill (Domfit F1. BH Fitness; Álava, Spain) and a MOTOmed. Before each session, heart rate and blood pressure were recorded. Participants then began a 5-minute warm up pedaling with no or little resistance. Then 20-minutes of continuous exercise was completed, where the study team member encouraged the participants to increase their work rate until they reached their individualized heart rate training zones (as described in the manuscript). Heart rate was monitored continuously using a wrist-worn pulsometer. If participants were not within their individualized training zones, the study team member would either encourage them to increase or decrease their work rate using verbal feedback. After 20-minutes, a 5-minute cool down was completed by pedaling with little or no resistance, similar to the warm-up. Participants then rested for 10-minutes in a seated position, after which heart rate and blood pressure were taken again to ensure they were returning towards baseline.

**Supplementary material S5** – distributional assumptions of the linear mixed effects models.

Two outcome measures (Trail-making Test Part A and Light physical activity) were log transformed for violating the normality of residual assumption. All Q-Q and residual vs fitted plots are found below. Plots show from left to right the Q-Q plot, the residual histogram and the residual vs fitted plots. The Kolmogorov-Smirnov statistic is also presented.
